# Variation in Accelerometer-Derived Instantaneous Acceleration Distribution Curves of Elite Male Soccer Players According to Playing Position: A Pilot Study

**DOI:** 10.3390/sports12090263

**Published:** 2024-09-23

**Authors:** Pedro Oliveira, Felipe Arruda Moura, Ivan Baptista, Fábio Yuzo Nakamura, José Afonso

**Affiliations:** 1 Centre of Research, Education, Innovation, and Intervention in Sport (CIFI2D), Faculty of Sport, University of Porto, 4200-450 Porto, Portugal; ivantm_@hotmail.com (I.B.); jneves@fade.up.pt (J.A.); 2 Laboratory of Applied Biomechanics, State University of Londrina, Londrina 86057-970, Brazil; felipemoura@uel.br; 3 Department of Computer Science, Faculty of Science and Technology, UiT The Arctic University of Norway, 9037 Tromso, Norway; 4 Research Center in Sport Sciences, Health Sciences and Human Development (CIDESD), University of Maia, 4475-690 Maia, Portugal; fnakamura@umaia.pt

**Keywords:** accelerometry, workload, team sports, match performance, physical load

## Abstract

The incorporation of triaxial accelerometers into Global Positioning Systems (GPS) has significantly advanced our understanding of accelerations in sports. However, inter-positional differences are unknown. This study aimed to explore the variability of acceleration and deceleration (Acc) distribution curves according to players’ positions during soccer matches. Thirty-seven male players from a national-level Portuguese club were monitored using 10 Hz GPS with an embedded accelerometer during the 2021/2022 season. Resultant Acc was obtained from the x (lateral), y (frontal/back), and z (vertical) axes and expressed in gravitational units (g). Statistical Parametric Mapping was employed to compare playing positions: central defenders (CD), fullbacks (FB), central midfielders (CM), wide midfielders (WM), and strikers (ST). All positions exhibited a decreasing Acc distribution curve, very similar in shape, with a high frequency of events in the lower ranges (i.e., 0 to 1 g) and a lower frequency of events in the higher values (2 to 10 g). Post hoc comparisons revealed significant differences between all positions, except between FB and WM. Out of 1000 points in the curve, CD had 540, 535, 414, and 264 different points compared to FB, CM, WM, and ST, respectively. These findings indicate that players in different positions face distinct demands during matches, emphasizing the need for position-specific Acc analysis and training programming. By analyzing Acc as a continuous variable, this study highlights the importance of individualized monitoring to ensure the comprehensive and precise tracking of all player activities, without overlooking or omitting critical information.

## 1. Introduction

Monitoring workload is crucial for athlete management [1] and helps practitioners and coaches with an objective framework for making evidence-based decisions [2], providing the ideal scenario to assess player fitness, fatigue, and readiness to train or play [3]. The total workload comprises training and match load, which can be categorized and quantified through internal and external measures [4]. Internal load refers to the psycho-physiological responses that occur during soccer training and competition [5]. These responses can be measured using objective methods, such as heart rate (HR), or subjective methods, such as the rating of perceived exertion (RPE) [6]. Meanwhile, external load is defined as the physical “work” performed by the player, which can be quantified using metrics obtained through technologies such as global positioning systems (GPS) [7].

The analysis of accelerations and decelerations is integral to workload quantification in various sports due to the accessibility of monitoring technologies such as GPS [8]. Acc corresponds to positive (acceleration) and negative (deceleration) changes in speed over time, respectively. These actions are important due to their high mechanical and metabolic demands, and they are repeatedly performed by players [9], being one of the most common actions preceding a goal situation [10]. As a result, they constitute a significant portion of the total load in training and match play [11]. Disregarding Acc could lead to an underestimation of match demands [12], which may result in inadequate preparation or insufficient recovery, compromising performance and increasing the risk of injury and illness [6].

Practitioners and researchers often classify Acc using arbitrary and non-consensual thresholds, typically without explanations for their use. The selection of these thresholds is often made at the practitioners and researchers’ discretion [13]. Additionally, these thresholds do not consider individual capacities and may provide an inaccurate representation of the relative intensity of acceleration tasks [14]. Determining a player’s maximum Acc to establish individual reference values could offer an alternative approach for monitoring Acc on an individual basis [15]. However, this method has some limitations, with studies employing threshold-based approaches for individualization, which may not reflect individual athletes’ physiological capacities or performance capabilities [12,16]. Moreover, Acc values are continuous variables, and statisticians have often advised against categorizing continuous variables due to the loss of information and precision [17]. In this sense, individual Acc analysis could help quantify the relative intensity of Acc tasks, acknowledging biological differences and physical capacities [14].

In line with a continuous analysis of Acc, analyzing Acc raw data curves from accelerometers, rather than relying solely on horizontal GPS-derived Acc, might be a more accurate solution for quantifying match and training load. The resultant Acc can be studied as the combination of Acc in the three axes of movement, with cumulative values used to quantify the workload throughout the analyzed session [18].

Players’ positions can also be investigated by analyzing the distribution curves of raw Acc data distribution curves. These curves may reveal distinct physical characteristics associated with different positions. For example, wide positions such as fullbacks (FB) and wide midfielders (WM) typically perform 10–20% more GPS-derived Acc efforts compared to central midfielders (CM) and central defenders (CD) [19]. In a study involving highly trained female soccer players from the Spanish first division, CD achieved the lowest values of Acc, while CM displayed the highest Acc values [20]. Conversely, the highest values of Acc were found in CD during the match day [21]. Thus, players may experience varying demands based on their positions.

Therefore, this pilot study aimed to investigate how the distribution curves of acceleration (Acc) vary according to players’ positions during official matches, as part of an exploratory approach to a new potential method. We analyzed the inter-positional variability of accelerometer-based Acc distribution curves. Based on previous studies, we hypothesized that players’ positions would present distinct acceleration distribution profiles, revealing position-specific competitive demands, particularly between CD and other positions.

## 2. Materials and Methods

### 2.1. Participants

**Figure 1:**
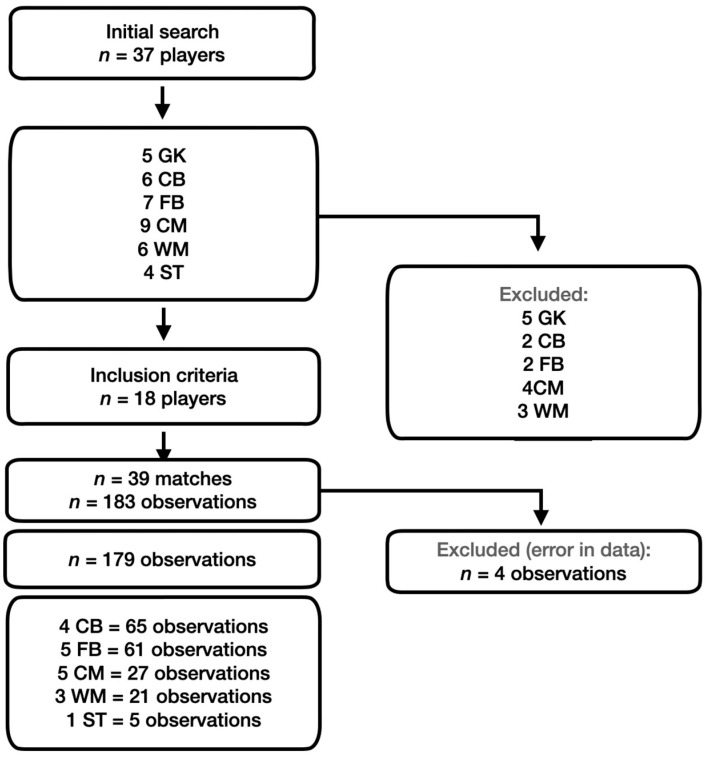
Flow diagram highlighting selection process of number of observations included in article.

A convenience sample was achieved due to the main author being part of the club’s technical staff. The sample consisted of 37 male players from a national-level soccer team in the top Portuguese division during the 2021/2022 season. Out of the total sample, 18 players met the inclusion criterion, which required each player to have participated in two or more full competitive matches, with a full match defined as playing 90 min (Figure 1). Goalkeepers were excluded from the analyses, and only male outfield players’ match data were retained. In total, 39 matches (34 from the national league and five from the national cups) and 179 playing actions (each representing one curve) were considered eligible for analysis. This included data from four CD, five FB, five CM, three WM, and one striker (ST). Each player participated in a minimum of five and a maximum of 25 matches, with an average of 9.9 ± 6.6 matches per player. The median number of matches was seven, with an interquartile range of five. Positions were further subdivided based on the hypothesis that each position might exhibit unique patterns in the Acc curves. While grouping positions more broadly may have enhanced statistical power, they could also have masked subtle differences, which are crucial for understanding the specific demands of each position during matches [22]. Consequently, the focus of this study was not on statistical power, as the primary objective was to test this new methodological approach. The data were collected retrospectively as part of the routine monitoring of team training and competition. The local Ethics Committee approved the study (CEFADE 49-2022).

### 2.2. Data Recording Procedures

The GPS (100 Hz, APEX Pro Series Apx + 4.14) units were turned on and secured to the upper back using a custom-made vest before the start of the warm-up (approximately 35 min prior to kick-off) to ensure optimal satellite lock [13]. Players wore the same device across multiple tracking sessions to minimize inter-unit variability bias [23].

At the end of each match, all GPS units were checked and placed in a docking system to download the data. Acc, corresponding to positive (acceleration) and negative (deceleration) changes in speed over time, respectively, were captured using Apex units’ accelerometers, which integrate GPS with a triaxial accelerometer. The Apex devices demonstrated suitable reliability for threshold-based Acc (coefficient of variation = 0.1 to 3.9%) [24]. The resultant acceleration vector magnitude as a function of time (i) (AVM), was obtained from x (lateral), y (frontal/back), and *z* axis (vertical) components (i.e., acx, acy, and acz, respectively), using the following formula [25]:

$$AVM\left(i\right)=\sqrt{(acx_{i+1}{-acx_{i})}^{2}+(acy_{i+1}{-acy_{i})}^{2}+(acz_{i+1}{-acz_{i})}^{2}}$$

All Acc data were expressed in gravitational units (g) where one gravitational unit equals the gravitational acceleration of 9.81 m/s^2^. The AVM is always expressed as a positive number, with the effect of gravity removed. The distribution of the data was explored across the 0 to 10 g range.

### 2.3. Statistical Analyses

Raw data were exported from the manufacturer’s software to MATLAB^®^ (R2022a 9.12.0) for processing. A histogram fit was performed with 1000 equally spaced points from a 0 to 10 g range, using a non-parametric Kernel-smoothing distribution to create curves of the resultant Acc. A Kernel distribution is recommended when a parametric distribution cannot properly describe the data or to avoid making assumptions about the data distribution [26]. Subsequently, each player (*n* = 179 observations) had a graph with 1000 points, where each point represented the frequency of the Acc values along the 0 to 10 g range.

Players were then divided into five groups according to their playing positions. Statistical analysis was conducted using Statistical Parametric Mapping (SPM) in MATLAB to compare players’ curves by their positions. SPM is specifically designed for the statistical analysis of time series and curves and has been previously applied to biomechanical time-series data in soccer, such as kicking, running, cutting movements, and landing techniques [27]. SPM one-way analysis of variance (ANOVA) with Bonferroni post hoc tests was used to identify significant differences between playing positions. The significance level was set at *p* ≤ 0.05. Effect sizes, which indicate the magnitude of differences between groups [28], were not calculated for each point on the 1000-point curve due to the complexity and standard practice in SPM analysis.

## 3. Results

**Figure 2:**
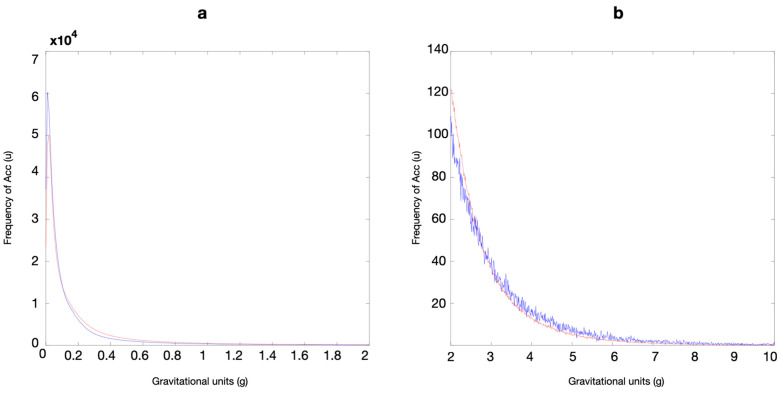
An example of one comparison between the average curve across all matches by one position against the average curve across all matches by another position (CD—red; FB—blue) with two x scales. (**a**) A chart for values between 0 and 2 g, with data smother for lower Acc curves. (**b**) A chart for values between 2 and 10 g and a reduced y-scale (0 to 140), with small peaks and valleys observed.

In this study of elite male soccer players, using a coarse-grained scale (i.e., 0 to 10 g), it was observed that all positions exhibited a similar decreasing curve. However, on a finer scale (between 0 and 2 g), the curves for different player positions frequently intersected. A high frequency of events was noted in the lower acceleration ranges (i.e., between 0 and 1 g), while fewer events were observed in the higher acceleration values. Data at the lower Acc were smoother compared to higher Acc. When visually expanding the regions of the curve with fewer occurrences (i.e., the lower part of the curves between 2 and 10 g, and with the y scale reduced between 0 and 140), a much noisier shape emerged. This was characterized by several peaks and valleys, indicating that the curves are not smooth when examined at a finer scale. Figure 2 presents an example of these curves (see Supplementary Materials).

**Figure 3:**
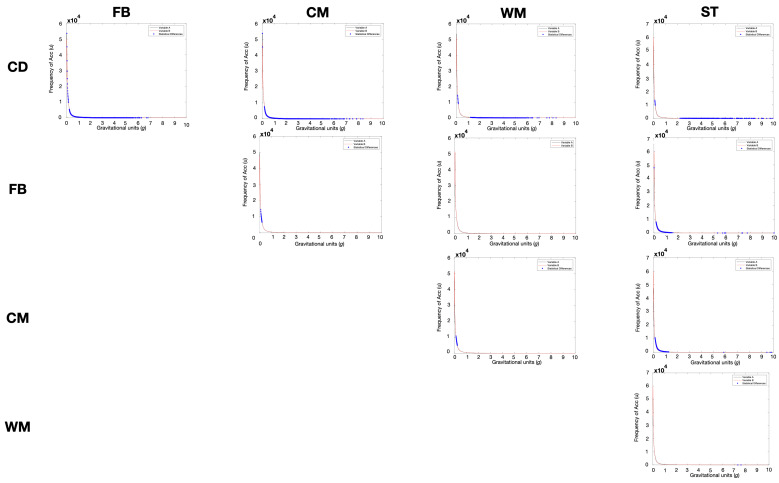
ANOVA with Bonferroni post hoc tests for resultant Acc with blue points representing number of statistically different points.

Figure 3 presents the ANOVA with Bonferroni post hoc tests to assess differences in resultant Acc between player positions. Despite the qualitative similarities observed in the curves, statistical differences were found between nearly all positions at certain points on the curve, with the exceptions of the comparisons between FB and WM, and between ST and WM, where differences were only observed at two points. The most significant differences are observed when comparing the CD position with others. Table 1 shows the number of different points between positions on a 1000-point scale. Upon inspecting the distribution curves, the CD have 540, 535, 414, and 264 points of difference compared to FB, CM, WM, and ST, respectively (Table 1).

## 4. Discussion

In this study, we aimed to conduct a pilot analysis of the Acc curves based on players’ positions during matches, with a focus on the inter-positional variability of raw data curves. We hypothesized that male players in different positions would exhibit distinct patterns in accelerometer-derived Acc curves, particularly highlighting differences between CD and other positions, with CD being exposed less frequently to Acc movements [29]. The results partially supported our hypothesis, showing significant differences between positions in some regions of the Acc curves. However, the overall shape of the curves was similar across positions.

All positions exhibited a similar decreasing curve, characterized by a high frequency of occurrences in the lower Acc range (0 to 1 g) and a lower frequency in the higher Acc range. This finding is consistent with previous research that observed a gradual decrease in frequency as Acc increased. For instance, in basketball players, it was reported that the frequency of movements decreased as the Acc thresholds increased: frequencies of 6455 movements were recorded with a resultant Acc of 4 g, 1745 movements with 6 g, and 466 movements with 8 g [30]. Similarly, in one study with young soccer players, 2649 movements were detected at resultant Acc thresholds of over 6 g and 798 movements at over 8 g [31]. Similar patterns were reported in elite rugby players, where the number of observed impacts decreased as g-forces increased, regardless of position [32]. Our results align with a recent systematic review, which found that lower Acc intensities occurred more frequently than higher Acc intensities [8]. The descending shape of the curves, with a predominance of low-intensity Acc values, can be attributed to common movements in locomotion such as walking, running, or rotating. These activities typically involve low Acc magnitudes and result in oscillations of the center of mass [33].

When inspecting the curves at a finer scale, particularly in the regions with fewer occurrences (between 2 and 10 g), we observed additional small peaks and valleys (see Figure 2b. The smoother appearance of the curves in lower Acc ranges contrasts with the noisier and more variable shape observed at higher Acc levels. This reflects the less frequent but more intense movements that players experience during matches [34].

Significant differences were noted at specific points along the 1000-point Acc curve scale. CD exhibited 540, 535, 414, and 263 points of difference compared to FB, CM, WM, and ST, respectively. These results indicate that 54% of the points on the 1000-point scale significantly differed between the CD and FB. Specifically, CD exhibited lower frequency values than other positions for Acc values between 2 and 10 g (Figure 2b and Figures S1–S3 in Supplementary Materials). This suggests that male players in different positions experience varying demands during matches. Specifically, CD are less exposed to high-intensity movements such as accelerating, decelerating, changing direction, or landing compared to other positions [29]. Previous research has identified CD as having the lowest GPS-derived Acc demands during matches [11]. However, our study used accelerometer-derived Acc for the comparisons. It remains to be investigated whether (at least part of) the observed differences are due to impacts or specific trunk movements.

Despite the observed differences, directly comparing our results with previous studies is challenging due to the varying categorizations of collisions and impact zones used across different studies, which often differ substantially. While some studies have sought to identify the movements requiring higher Acc by examining movements that generate >4, >6, and >8 g [31,35,36], others have classified collisions and impacts into five [33] or six distinct zones [32]. Our study supports the notion that Acc should be analyzed as a continuous variable. Analyzing Acc continuously may be more appropriate for understanding individual demands and avoiding information loss associated with categorization. To our knowledge, previous studies have individualized Acc based on each player’s maximum Acc, but they have used GPS-derived accelerations rather than raw accelerometer data.

It is important to acknowledge the limitations of the current study. First, the results might be influenced by players’ characteristics and their specific tactical roles during the matches. Different tactical systems and player distributions in soccer could affect the value of Acc observed [37]. Second, the relatively small number of observations for ST and WM (5 and 21, respectively) might have affected the results. Thus, the replication of this pilot study with a larger sample size, capable of providing sufficient statistical power, is recommended. Third, our study did not attempt to identify which specific movements generated the highest Acc values during a match, unlike some previous studies [31,36]. Additionally, our results were measured using accelerometers placed on the upper back, which are subject to shock attenuation. As a result, some of the Acc impacts experienced at the feet level may be dissipated by the musculo-skeletal system [38]. The number of acquired satellites and the horizontal dilution were not recorded during data collection, which may reduce the reliability of the results.

While this pilot study provides initial insights into position-specific Acc demands, supporting the initial hypothesis, future studies should expand on this by including a larger sample size, a broader range of positions, and different tactical setups. Additionally, a more detailed analysis of the types of movements associated with high Acc values could further refine our understanding of position-specific demands. Future research should also explore collisions and impacts using gravitational units, as the literature presents divergent thresholds, and investigate how these vary across different phases of matches. Additionally, careful attention should be given to data processing methods, as different techniques can alter the summary metrics of Acc data [39,40]. Investigating the context and types of movements associated with high Acc could provide further insights into positional demands and assist in refining training strategies.

## 5. Conclusions

Despite similar distribution curve shapes, we identified significant between-position differences in accelerometer Acc distribution curves, particularly between CD and other positions. The high frequency of lower Acc values suggests that common actions such as walking, running, and slight directional changes are predominant, while higher Acc values are less frequent. These results reinforce the importance of examining the specific Acc demands for each positional role in male soccer players. However, future studies must ensure adequate statistical power. Coaches and practitioners should design training programs that reflect the unique Acc requirements of each position. For instance, CD may benefit from less emphasis on high-intensity acceleration drills compared to FB or CM, who experience higher-intensity actions more frequently. Analyzing Acc as an individual and continuous variable, rather than categorically, may be a more suitable approach for monitoring an athlete’s specific demands. This method ensures comprehensive and precise tracking of all player activities, without overlooking or omitting any important information.

## Figures and Tables

**Table 1:** Number of different points (in 1000-point curve) between positions.

	FB	CM	WM	ST
CD	540	535	414	264
FB		12	0	129
CM			12	109
WM				2

## Data Availability

The original contributions presented in the study are included in the article/Supplementary Materials, further inquiries can be directed to the corresponding author.

## Supplementary Materials

The following supporting information can be downloaded at https://www.mdpi.com/article/10.3390/sports12090263/s1, Figure S1: An example of one comparison between the average curve across all matches by one position against the average curve across all matches by another position (CD—red; CM—blue) with two x scales. (**a**) A chart for values between 0 and 2 g, with data smother for lower Acc curves. (**b**) A chart for values between 2 and 10 g and a reduced y-scale (0 to 120), with small peaks and valleys observed; Figure S2: An example of one comparison between the average curve across all matches by one position against the average curve across all matches by another position (CD—red; WM—blue) with two x scales. (**a**) A chart for values between 0 and 2 g, with data smother for lower Acc curves. (**b**) A chart for values between 2 and 10 g and a reduced y-scale (0 to 140), with small peaks and valleys observed. Figure S3: An example of one comparison between the average curve across all matches by one position against the average curve across all matches by another position (CD—red; ST—blue) with two x scales. (**a**) A chart for values between 0 and 2 g, with data smother for lower Acc curves. (**b**) A chart for values between 2 and 10 g and a reduced y-scale (0 to 120), with small peaks and valleys observed.
