# Supplementary material for: Variation in Accelerometer-Derived Instantaneous Acceleration Distribution Curves of Elite Male Soccer Players According to Playing Position: A Pilot Study
Source: Sports (Basel). 2024 Sep 23;12(9):263. doi: 10.3390/sports12090263 (PMC11436242; doi:10.3390/sports12090263)
Supplement: Supplementary file 1 [file sports-12-00263-s001.zip › sports-3120947-supplementary.pdf]

Supplementary material

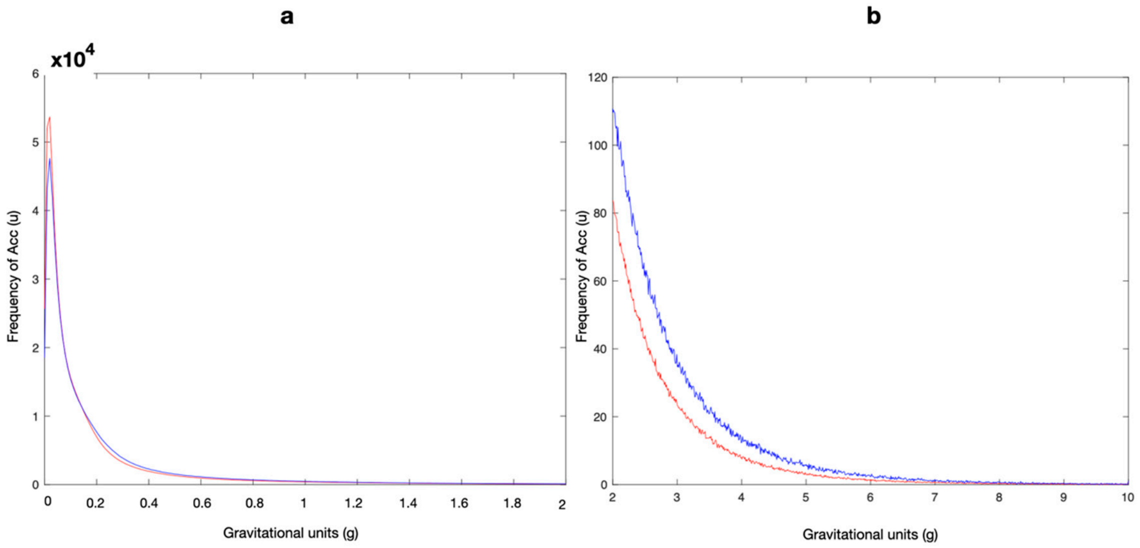


**Figure S1. An** example of one comparison between the average curve across all matches by one position against the average curve across all matches by another position (CD—red; CM—blue) with two x scales. (**a**) A chart for values between 0 and 2 g, with data smother for lower Acc curves. (**b**) A chart for values between 2 and 10 g and a reduced y-scale (0 to 120), with small peaks and valleys observed.

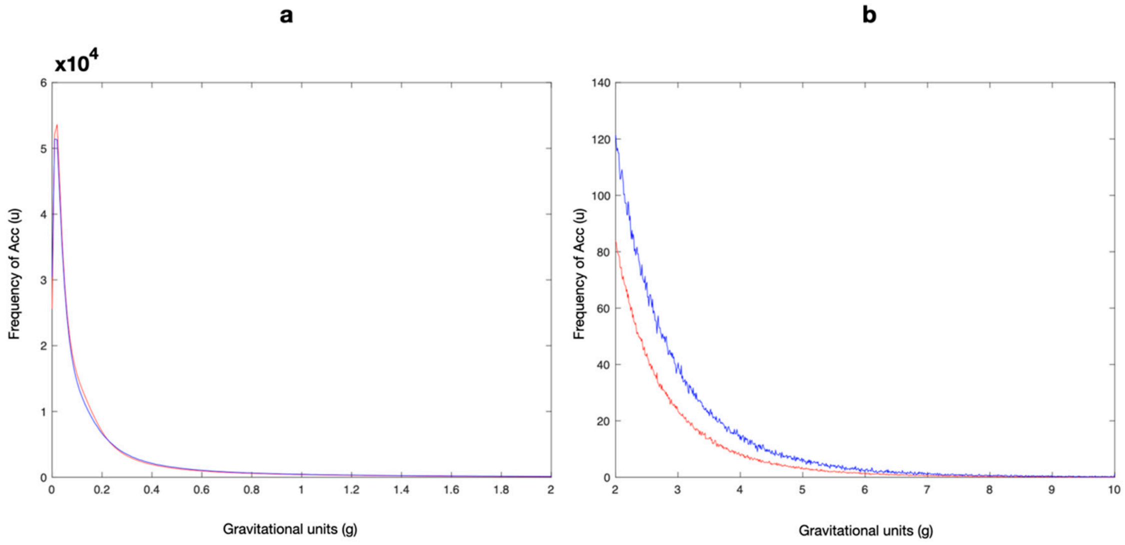


**Figure S2. An** example of one comparison between the average curve across all matches by one position against the average curve across all matches by another position (CD—red; WM—blue) with two x scales. (**a**) A chart for values between 0 and 2 g, with data smother for lower Acc curves. (**b**) A chart for values between 2 and 10 g and a reduced y-scale (0 to 140), with small peaks and valleys observed.

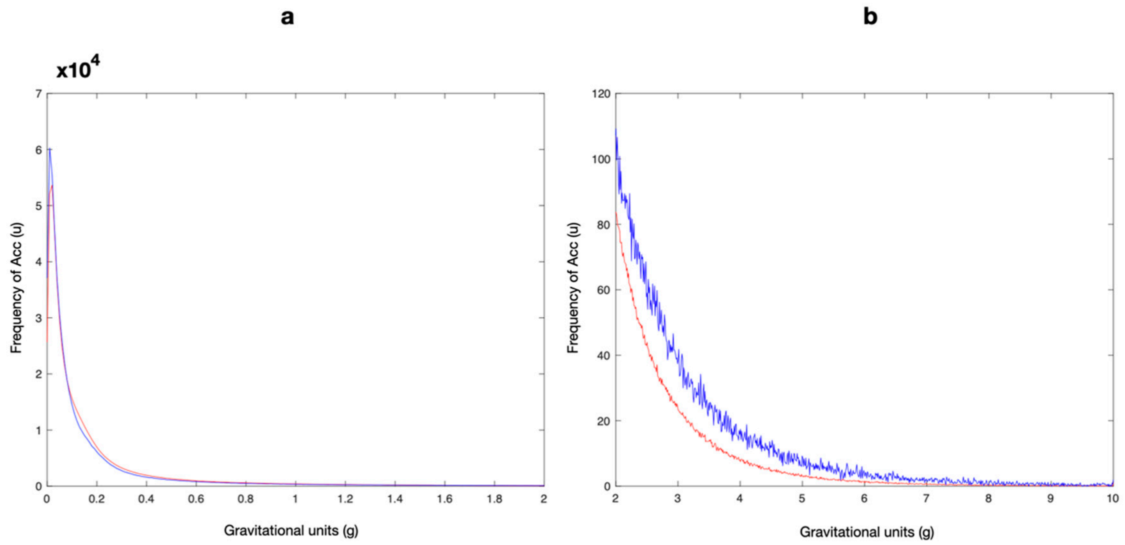


**Figure S3. An** example of one comparison between the average curve across all matches by one position against the average curve across all matches by another position (CD—red; ST—blue) with two x scales. (**a**) A chart for values between 0 and 2 g, with data smother for lower Acc curves. (**b**) A chart for values between 2 and 10 g and a reduced y-scale (0 to 120), with small peaks and valleys observed.
